# End-to-End Deep Learning of Non-rigid Groupwise Registration and Reconstruction of Dynamic MRI

**DOI:** 10.3389/fcvm.2022.880186

**Published:** 2022-04-28

**Authors:** Junwei Yang, Thomas Küstner, Peng Hu, Pietro Liò, Haikun Qi

**Affiliations:** ^1^Department of Computer Science and Technology, University of Cambridge, Cambridge, United Kingdom; ^2^The School of Biomedical Engineering, ShanghaiTech University, Shanghai, China; ^3^Medical Image and Data Analysis (MIDAS.lab), Department of Diagnostic and Interventional Radiology, University Hospital of Tübingen, Tübingen, Germany

**Keywords:** dynamic MR imaging, deep learning, reconstruction, registration, multi-task learning

## Abstract

Temporal correlation has been exploited for accelerated dynamic MRI reconstruction. Some methods have modeled inter-frame motion into the reconstruction process to produce temporally aligned image series and higher reconstruction quality. However, traditional motion-compensated approaches requiring iterative optimization of registration and reconstruction are time-consuming, while most deep learning-based methods neglect motion in the reconstruction process. We propose an unrolled deep learning framework with each iteration consisting of a groupwise diffeomorphic registration network (GRN) and a motion-augmented reconstruction network. Specifically, the whole dynamic sequence is registered at once to an implicit template which is used to generate a new set of dynamic images to efficiently exploit the full temporal information of the acquired data via the GRN. The generated dynamic sequence is then incorporated into the reconstruction network to augment the reconstruction performance. The registration and reconstruction networks are optimized in an end-to-end fashion for simultaneous motion estimation and reconstruction of dynamic images. The effectiveness of the proposed method is validated in highly accelerated cardiac cine MRI by comparing with other state-of-the-art approaches.

## 1. Introduction

Dynamic MRI has found various applications in clinical practice, such as cardiovascular, pulmonary and abdominal imaging. Rapid data acquisition is required in dynamic MRI to provide sufficient spatial and temporal resolution. However, MRI is known to have low acquisition speed, and accelerated data acquisition and reconstruction is an important topic in dynamic MRI.

Parallel imaging (1, 2) and compressed sensing (CS) (3) have achieved great success in accelerating MRI by recovering images from undersampled measurements that are fewer than those required by the Nyquist law. Image reconstruction from undersampled MRI data is an ill-posed inverse problem and regularization on prior information is required to stabilize the solution. For dynamic MRI reconstruction, regularizations have been designed to exploit the spatial and temporal correlation of dynamic images, including sparsity regularization in transformed domains (4–6), low-rank constraint (7), or the combination of sparsity and low-rank priors (8–10).

Attempts have been made to further improve the reconstruction performance through modeling the motion to increase the signal sparsity, which is achieved by exploiting the abrupt signal changes through time caused by inter-frame motion (11–20). The challenge of applying motion estimation (ME) and motion compensation (MC) to dynamic MRI reconstruction is the lack of good quality dynamic images for motion estimation. In k-t FOCUSS with ME/MC (12), each frame is individually registered to a high-quality reference image using a block matching algorithm. However, reference images of good quality are usually not available. The MASTeR algorithm (13) estimates motion between adjacent frames to construct motion-adaptive regularization. Some authors have modeled the reconstruction process as a joint task of motion estimation and reconstruction (19–21), with the two tasks being optimized alternatively, such as MC-JPDAL (19) where the dynamic sequences and the inter-frame motion vectors are estimated jointly by combining an intensity-based optical flow constraint with the traditional CS scheme, and then the reconstructed dynamic images can be further refined with the estimated motion vectors.

However, pairwise registration is performed in the above mentioned approaches, involving only two frames for ME, and consequently additional information in the rest of the frames cannot be exploited, which makes ME sensitive to undersampling artifacts in the dynamic images. To overcome such shortcomings of pairwise ME, Royuela-del Val et al. (16) propose to use groupwise non-rigid registration to register the full set of dynamic images for only once to generate a temporally-aligned dynamic sequence to improve the dynamic reconstruction in a CS framework (GW-CS). Overall, ME/MC can be integrated into the dynamic reconstruction procedure to improve the sparsity of CS reconstruction or provide additional constraints to improve the reconstruction performance. However, the iterative optimization of ME and CS reconstruction is computationally demanding. Especially, the non-rigid registration for ME requires iterative optimization which takes up a lot of the computation time. Although methods have been developed to reduce the non-rigid registration time from hours to minutes, the computation time of registering a sequence of dynamic images using groupwise registration or multiple pairwise registrations can still be considerable. Moreover, the non-rigid registration step has to be performed several times in the motion-compensated reconstruction procedure, so the traditional motion-compensated reconstruction approaches tend to be time-consuming.

Deep learning-based MRI reconstruction methods have been proposed to significantly reduce the reconstruction time and have demonstrated better reconstruction quality than CS-based methods. Deep learning approaches are usually designed to learn the mapping from undersampled images/measurements to fully sampled images/measurements based on the training data (22). Whilst most deep learning reconstruction methods are for static images, networks such as 3D convolutional neural network, 2D/3D+1D convolutional networks and 2D recurrent neural network have been proposed for dynamic reconstruction (23–28). Those state-of-the-art methods can be impaired by spatially unmatched anatomies as they could lead to blurry or temporal inconsistent images for highly undersampled data.

However, so far, only limited works have incorporated motion information into deep learning-based reconstruction. Previously, we have developed an end-to-end trainable framework for motion corrected 3D cardiac image reconstruction (29), but this framework is not applicable to dynamic reconstruction. Huang et al. develop a motion-guided dynamic reconstruction network that utilizes motion estimation and motion compensation to improve the reconstruction quality, which, however, requires a fully sampled reference frame that may not be available (30). The most relevant work is Seegoolam et al. (31), where motion is estimated in each cascade from an intermediate reconstructed image to fuse the full information of acquired data and to aid in improving reconstruction performance. However, this method requires a large number of pairwise registrations to estimate the motion between a specific frame and all the other frames, which are computationally redundant and expensive. A more efficient motion estimation framework is yet to be developed and to be incorporated into the dynamic reconstruction network.

To this end, we propose a novel joint learning approach that performs non-rigid groupwise registration and reconstruction of highly undersampled dynamic MRI. An unrolled deep learning architecture is constructed with each unrolled iteration consisting of a groupwise diffeomorphic registration network (GRN) and a reconstruction network. The GRN is used to efficiently exploit the dynamic information across all frames by estimating the invertible motion fields between the whole sequence and an implicit template, thereby generating a new set of dynamic images by transforming the template with estimated motion fields. The motion-augmented dynamic sequence is then incorporated into the reconstruction network to improve the reconstruction performance. For GRN, we employ the self-supervised deep learning registration model, which is more efficient and robust than traditional motion estimation algorithms in the presence of undersampling artifacts (32, 33). To the best of our knowledge, this is the first work that embeds groupwise registration network into the deep learning reconstruction framework to exploit the full temporal information of the acquired data to aid in the dynamic MRI reconstruction. The contributions of this work are three aspects. Firstly, we design a groupwise diffeomorphic registration network that provides invertible motion fields, and requires no motion ground truth for training and is robust to undersampling artifacts. Secondly, we systemically compare the performance of groupwise registration and pairwise registration in the proposed joint learning approach regarding motion estimation and reconstruction performance. Finally, we devise a composite loss which comprises of a motion estimation loss and an image reconstruction loss to train the joint learning network on an end-to-end basis. The effectiveness of the proposed method is validated in highly accelerated cardiac cine MRI by comparing with other state-of-the-art non-learning-based and learning-based dynamic MRI reconstruction methods.

## 2. Materials and Methods

The proposed groupwise registration and dynamic reconstruction network (GRDRN) consists of several unrolled iterations with each iteration consisting of a groupwise registration network (GRN) to generate motion-augmented dynamic sequence and a dealiasing reconstruction network. Details of each part is introduced as follows.

### 2.1. Learning-Based Groupwise Registration

Provided a set of dynamic images *X* = {*X*_1_, ⋯ , *X*_*N*_} with *N* being the number of dynamic frames, the groupwise registration attempts to simultaneously estimate a set of transformations *T* = {*T*_1_, ⋯ , *T*_*N*_} that warp the images *X* to a common reference image $\bar{X}$, such that the deformed image *T*_*m*_ ∘ *X*_*m*_ is similar to *T*_*n*_ ∘ *X*_*n*_ ∀*m* ≠ *n* with ∘ being the warping operator. To obtain differentiable and invertible deformation fields, and following the conventional diffeomorphic registration that integrates stationary or time-varying velocity fields (34), a set of stationary velocity fields υ = {*v*_1_, ⋯ , *v*_*N*_}, pointing from the template image $\bar{X}$ to the dynamic images *X* are estimated. Subsequently, the transformations *T* and their inverse transformations $T^{-1}=\left\{T_{1}^{-1},\cdots \;,T_{N}^{-1}\right\}$ can be estimated by respectively integrating the velocity field υ and the negative velocity field −υ over unit time (34, 35).

**Figure 1:**
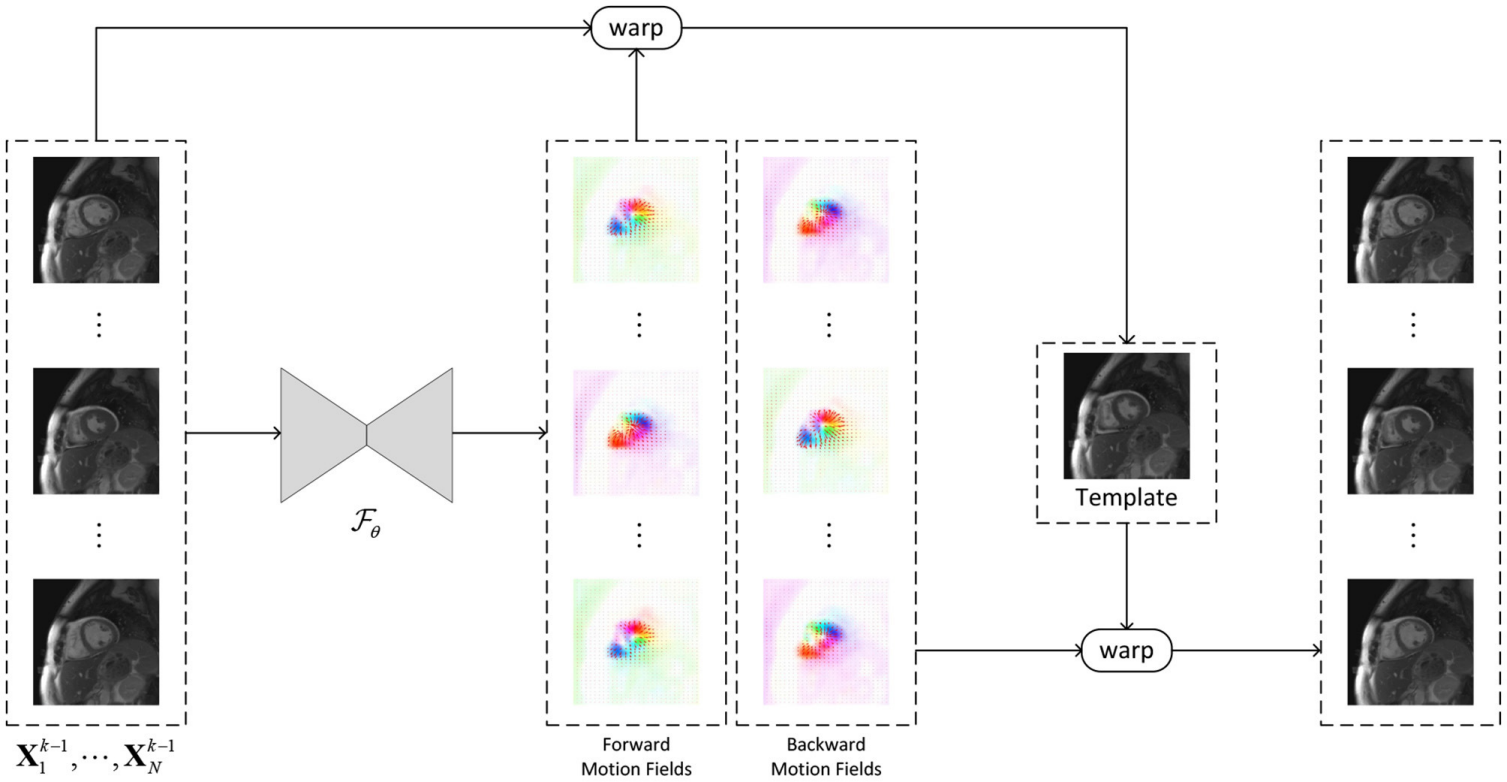
The framework of the learning-based groupwise registration network (GRN, $F_{\theta }$). The GRN takes a sequence of images as input and generates the invertible forward and backward motion fields between an implicit template and the dynamic images, where the template is the average of the warped dynamic images with the forward motion. A new sequence of dynamic images can be generated by warping the template using the backward motion fields.

For learning-based groupwise registration (Figure 1), we define a network $F_{\theta }$ with parameters of θ to simultaneously estimate a set of transformations for a dynamic sequence: $\upsilon =F_{\theta }\left(X\right)$ and the transformations *T* is constructed based on υ. The network is optimized via the intensity-based similarity (mean-squared-error, MSE) and velocity field smoothness loss:

(1) $$\begin{array}{l} L_{\text{reg}}\left(X,T\right)=\frac{1}{N}\overset{N}{\underset{n=1}{\sum }}||\bar{X}-T_{n}\circ X_{n}|{|}_{2}^{2}+\frac{1}{N}\overset{N}{\underset{n=1}{\sum }}||X_{n}-T_{n}^{-1}\circ \bar{X}|{|}_{2}^{2}\\ \;+\alpha \frac{1}{N}\overset{N}{\underset{n=1}{\sum }}||\nabla v_{n}|{|}_{2}^{2}, \end{array}$$

where the first term enforces the similarity between the implicit reference image and the warped dynamic images; the second term imposes the intensity similarity between the original dynamic images and the generated dynamic images by transforming the template with the inverse transformations ($T_{n}^{-1}\circ \bar{X}$); the third term is to encourage the smoothness of the estimated velocity fields and α is the regularization weight. The implicit template image is defined as the average of the warped dynamic images (36, 37):

(2) $$\begin{array}{r} \bar{X}=\frac{1}{N}\overset{N}{\underset{n=1}{\sum }}T_{n}\circ X_{n}. \end{array}$$

In addition, to enforce the reference image to lie in the geometric center of the group, as proposed in Li et al. (37), the average velocity field is subtracted from each of the estimated velocity fields: ${\hat{v}}_{n}=\frac{1}{N}\sum_{n=1}^{N}v_{n}$, and the sum of all the velocity fields is consequently zero.

### 2.2. Motion-Augmented Dynamic Reconstruction

**Figure 2:**
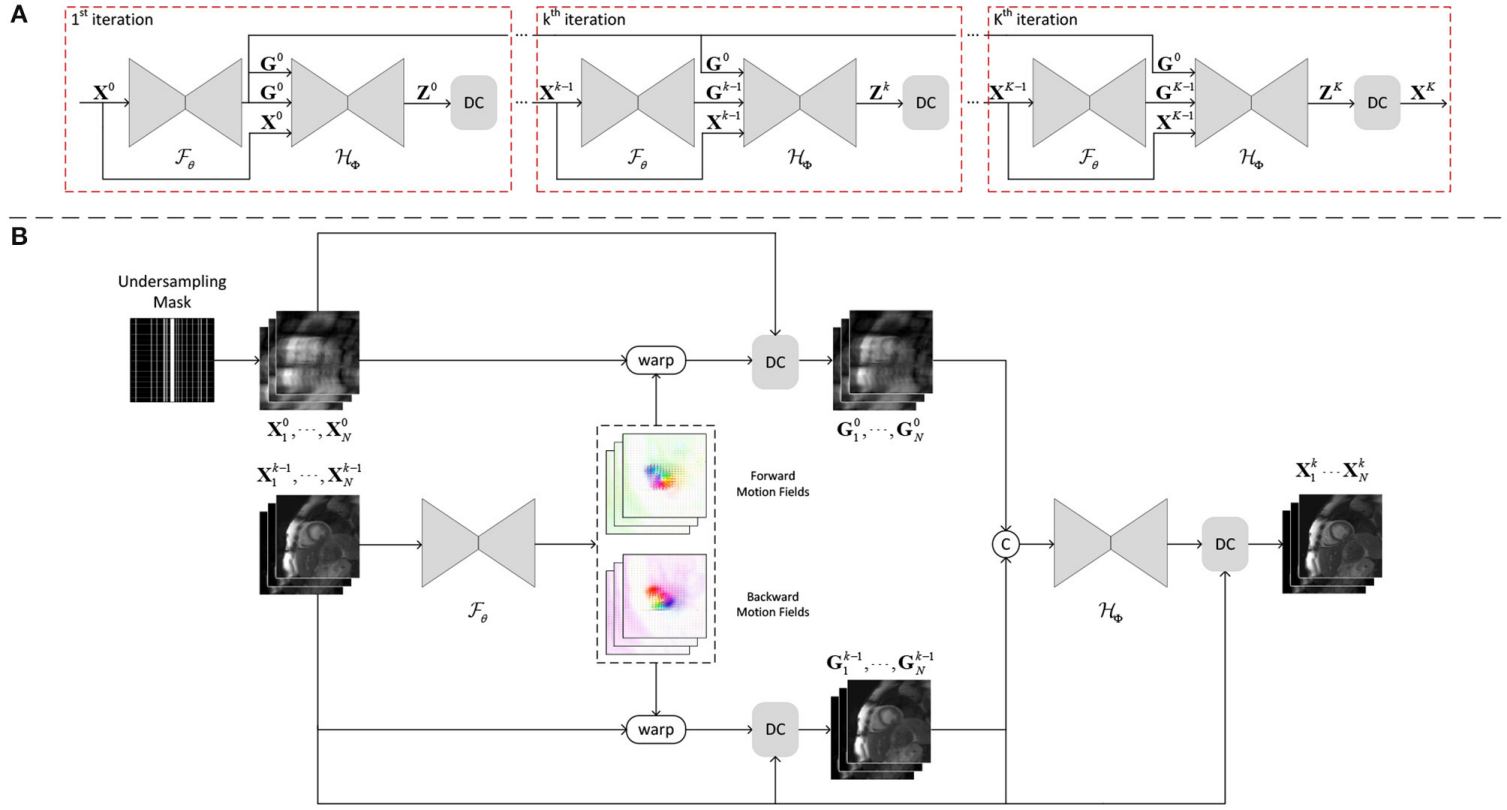
**(A)** The unrolled architecture of GRDRN for K iterations, where $F_{\theta }$ and $H_{\Phi }$ is the groupwise registration and dynamic reconstruction network respectively. **(B)** The detailed framework of the *k*-th iteration in GRDRN. The groupwise registration network $F_{\theta }$ takes as input the reconstructed dynamic images from the previous iteration and outputs the invertible motion fields between the implicit template and dynamic images. The reconstruction network $H_{\Phi }$ denoises the dynamic images with additional inputs of the motion augmented dynamic sequences. After the data consistency layer (DC), the denoised dynamic images will be inputted to the next iteration.

We aim to integrate the groupwise registration module, GRN, into an unrolled dynamic MRI reconstruction network (Figure 2), where the registration and reconstruction modules are optimized iteratively. In each iteration, the GRN takes as input the magnitude of undersampled or intermediate reconstructed dynamic images and outputs the velocity fields between the implicit template and the dynamic images, from which the sets of transformations and inverse transformations can be obtained. As defined in Equation (2), the implicit template image is the average of all the warped dynamic images so that the full information of dynamic measurements is efficiently fused after motion compensation. Then, a set of dynamic images can be generated by warping the template image ${\bar{X}}^{k}$ with the inverse transformations $T^{k-1}=\left\{T_{1}^{k-1},\cdots \;,T_{N}^{k-1}\right\}$ at the *k*-th iteration:

(3) $$\begin{array}{r} G_{n}^{k}=\text{DC}\left(T_{n}^{k-1}\circ {\bar{X}}^{k}\right), \end{array}$$

where $G^{k}=\left\{G_{1}^{k},\cdots \;,G_{N}^{k}\right\}$ is the generated, motion-augmented dynamic images, DC indicates data consistency enforcement which is performed by plugging in the originally acquired data for each frame. The zero-filled reconstructed images are denoted as *X*^0^, and the corresponding template image and generated dynamic images are ${\bar{X}}^{0}$ and *G*^0^, respectively.

The motion-augmented dynamic image series which has fused the information along the temporal dimension is used as the input to the dealiasing network to aid the reconstruction. Specifically, two sets of motion-augmented dynamic images are generated with, respectively, the output of the previous iteration and the zero-filling reconstructed images and are stacked together as the input to the reconstruction network, the combination of which was demonstrated to be effective as in Seegoolam et al. (31). Therefore, in the *k*-th iteration, the dealiased dynamic images *Z*^*k*^ are:

(4) $$\begin{array}{r} Z^{k}=H_{\Phi }\left(G^{k-1},G^{0},X^{k-1}\right)+X^{k-1}, \end{array}$$

where the residual learning strategy is employed and $H_{\Phi }$ is the dealiasing network patameterized by Φ. The output of the dealiasing network goes through the DC layer (23) to obtain the reconstruction at the *k*-th iteration: *X*^*k*^ = DC(*Z*^*k*^), which are applied as the input to the next iteration for groupwise registration and motion-augmented reconstruction.

### 2.3. End-to-End Optimization Framework

The registration loss and reconstruction loss are designed for each unrolled iteration to train the proposed GRDRN. For registration, previous works (32, 33) have demonstrated that by constructing the MSE loss based on the fully sampled ground truth images, it is possible to learn the motion from undersampled images. Therefore, while the input to GRN is the undersampled or intermediate reconstructed images, the registration loss as defined in Equation (1) for the *k*-th iteration $L_{\text{reg}}^{k}\left(X^{\text{gt}},T^{k}\right)$ is calculated based on the fully sampled ground truth dynamic images $X^{\text{gt}}=\left\{X_{1}^{\text{gt}},\cdots \;,X_{N}^{\text{gt}}\right\}$. The reconstruction of each unrolled iteration is compared with the fully sampled ground truth to calculate the reconstruction loss: $L_{\text{rec}}^{k}\left(X^{\text{gt}},X^{k}\right)=\frac{1}{N}\sum_{n=1}^{N}||X_{n}^{\text{gt}}-X_{n}^{k}|{|}_{2}^{2}$. The joint training loss of each iteration is a weighted combination of the registration loss and reconstruction loss, and the end-to-end optimization problem is formulated as:

(5) $$\begin{array}{r} \underset{\theta ,\Phi }{arg min}\overset{K}{\underset{k=1}{\sum }}w^{k}\left(L_{\text{rec}}^{k}\left(X^{\text{gt}},X^{k}\right)+\lambda L_{\text{reg}}^{k}\left(X^{\text{gt}},T^{k}\right)\right), \end{array}$$

where *K* is the number of unrolled iterations, *w*_*k*_ = exp(*k* − *K*) is the weighting factor of each unrolled iteration, and λ is the weight controlling the contribution of the registration loss. It is noted that the network parameters are shared for different unrolled iterations to reduce the number of trainable parameters (38).

The UNet architecture (39) is adapted for the registration network $F_{\theta }$ and dealiasing network $H_{\Phi }$, while the network architecture can be modified for specific applications. We adopt the 2D UNet for $F_{\theta }$ with the magnitude of the dynamic images stacked along the channel dimension, and employ the 3D UNet for $H_{\Phi }$ with the real and imaginary components of the complex images stacked along the channel dimension. The convolution layers produce a set of *C* feature maps by individually convolving the input with *C* kernels. In this work, we use *C* = [32, 64, 128, 256, 128, 64, 32] for both $F_{\theta }$ and $H_{\Phi }$. Each convolution layer is of kernel size (3, 3) and (3, 3, 3) for $F_{\theta }$ and $H_{\Phi }$ respectively, followed by a leaky ReLU layer for nonlinear activation except for the last convolution layer where no activation function is used. Max-pooling and transposed convolution is respectively used for the downscale and upscale layers. The whole model has a total number of around 7M trainable parameters.

### 2.4. Experiments

We evaluate our method on the dataset of breath-held 2D cardiac cine MRI, where repeated breath-holds are usually required to cover the whole left ventricle. During the acquisition, acceleration is essential to reduce the scan time and the number of breath-holds, and highly accelerated MRI acquisition may enable the scan of whole-heart cine in a single breath-hold. We therefore target for high acceleration factors of 8×, 12×, and 16×. We evaluate the motion estimation and reconstruction performance of the proposed method in retrospectively undersampled cardiac cine MRI data. We compare GRDRN with state-of-the-art conventional dynamic reconstruction methods with ME/MC and learning-based dynamic reconstruction approaches with pairwise registration or without motion estimation. The compared methods are detailed in the Section of 2.4.3.

#### 2.4.1. Dataset and Preprocessing

We use a dataset of 56 cardiac cine MRI scans including 34 healthy volunteers and 22 patients with suspected cardiovascular diseases acquired using a commercially available 2D balanced steady-state free precession cine imaging technique. Multiple short-axis slices are acquired in 6–8 breath-holds of around 12-s duration (2 slices per breath-hold) each with 20 s pause in between, which results in an acquisition time of 3–4 min. The imaging parameters are: in-plane spatial resolution = 1.9 × 1.9 mm; matrix size = 176 × 144; slice thickness = 8 mm; TR/TE = 2.12/1.06; flip angle = 52°; bandwidth = 915 Hz/pixel; 25 dynamic frames with temporal resolution of 40 ms; parallel imaging factor = 2.

The 2-fold accelerated data is firstly reconstructed using a k-space based parallel imaging method GRAPPA (2). The reconstructed multi-coil k-space data is coil combined into single-coil k-space data and regarded as the “fully sampled” reference in this work. The image reconstructed by inverse Fourier Transform of the single-coil k-space is thus considered as the ground truth image for training and evaluation of the reconstruction methods. We have randomly selected 35 subjects for training, 3 subjects for validation and 18 subjects for testing. For each subject, 6–8 central slices are selected resulting in 263, 23, and 128 slices for training, validation and testing, respectively.

We consider Cartesian undersampling in this work, where the data is fully sampled along the frequency-encoding dimension and is randomly undersampled along the phase-encoding dimension. The sampling density conforms to a zero-mean Gaussian distribution, and five central k-space lines are always sampled for each frame. We follow the implementation in Schlemper et al. (23) to generate undersampling masks for 8×, 12×, and 16× acceleration factors.

#### 2.4.2. Implementation Details

The number of unrolled iterations in the proposed GRDRN is set to 4. The weighting factor α and λ was, respectively, optimized to be 0.05 and 1 by a limited number of searches. The network performance reaches a plateau within 60 epochs. The training samples are shuffled at the beginning of each epoch and the undersampling masks are generated on-the-fly during training to reduce overfitting. We train the network with Adam optimizer with the initial learning rate of 1*e* − 4, which is reduced by half every 20 epochs. The network is trained on a single NVIDIA GeForce RTX 3090 graphics card. With batch size of 1, the network training took around 12 h and 19 GB GPU memory.

#### 2.4.3. Baseline Methods

The reconstruction performance of the proposed method is compared with state-of-the-art non-learning-based and learning-based dynamic MRI reconstruction methods. Specifically, for conventional methods, we consider two reconstruction approaches that modeled cardiac motion during reconstruction. One is GW-CS (16), which adopts a B-spline based groupwise registration approach to register the whole sequence to a common template to generate temporally aligned dynamic images, which are highly sparse in temporally transformed domains and can benefit the compressed sensing reconstruction. The other is MC-JPDAL (19) which combines intensity-based optical flow constraint with the compressed sensing scheme to jointly reconstruct the dynamic sequence and estimate the motion fields between adjacent frames. Then, the dynamic reconstruction is further refined through motion compensation with the estimated motion fields in MC-JPDAL. We have used the codes provided by the authors: GW-CS, https://www.lpi.tel.uva.es/node/609; MC-JPDAL, https://github.com/ning22/Motion-Compensated-Dynamic-MRI-Reconstruction-with-Local-Affine-Optical-Flow-Estimation. The relevant reconstruction parameters are optimized for our dataset.

For the learning-based methods, the motion-estimation groupwise registration network is removed from the joint learning approach to understand its benefits. This thus results in a classic unrolled deep learning reconstruction framework which alternates between network-based dealiasing and data consistency enforcement (23), which is termed as CNN-DC in this work. Furthermore, we hypothesize that the groupwise registration should perform better than pairwise registration in registering a group of dynamic images. To validate this point, we replace the groupwise registration network with a pairwise registration network where the dynamic images are registered to a selected frame instead of the learned implicit template. For the considered cardiac cine MRI, the diastolic phase which is the first frame in the dynamic sequence is selected as the registration reference. The pairwise registration-based motion estimating dynamic reconstruction framework is called PRDRN in this work. Other components are kept the same for GRDRN and PRDRN except for the registration scheme. The CNN-DC and PRDRN are trained with the same data and training settings to GRDRN for fair comparison.

#### 2.4.4. Evaluations

We analyze the reconstruction performance quantitatively by calculating the peak signal-to-noise ratio (PSNR) and the image structure similarity (SSIM) (40) between the ground truth images and the reconstructed images with each reconstruction method.

We evaluate the motion estimation performance for the pairwise and groupwise registration adopted in the deep learning dynamic reconstruction task, PRDRN and GRDRN. The motion estimation error cannot be calculated directly as motion ground truth is not available. We propose to evaluate the registration performance by evaluating the similarity between the generated dynamic images with estimated motion fields and the original dynamic ones. All learning-based registration methods in this work are able to produce invertible motion fields, where backward motion fields point from the dynamic images to the reference image, and forward motion fields point from the reference image to the dynamic images. For GRDRN, we use the estimated forward motion fields to warp the fully sampled dynamic images to obtain the implicit template, and then warp the template image with the backward motion fields to generate a new set of dynamic images. For PRDRN, we warp the designated reference image with the backward motion for the motion augmented dynamic images. We then calculate PSNR and SSIM between the generated dynamic images and the original fully sampled dynamic images with the assumption that better registration performance should result in higher PSNR and SSIM metrics.

## 3. Results

### 3.1. Dynamic Reconstruction

**Figure 3:**
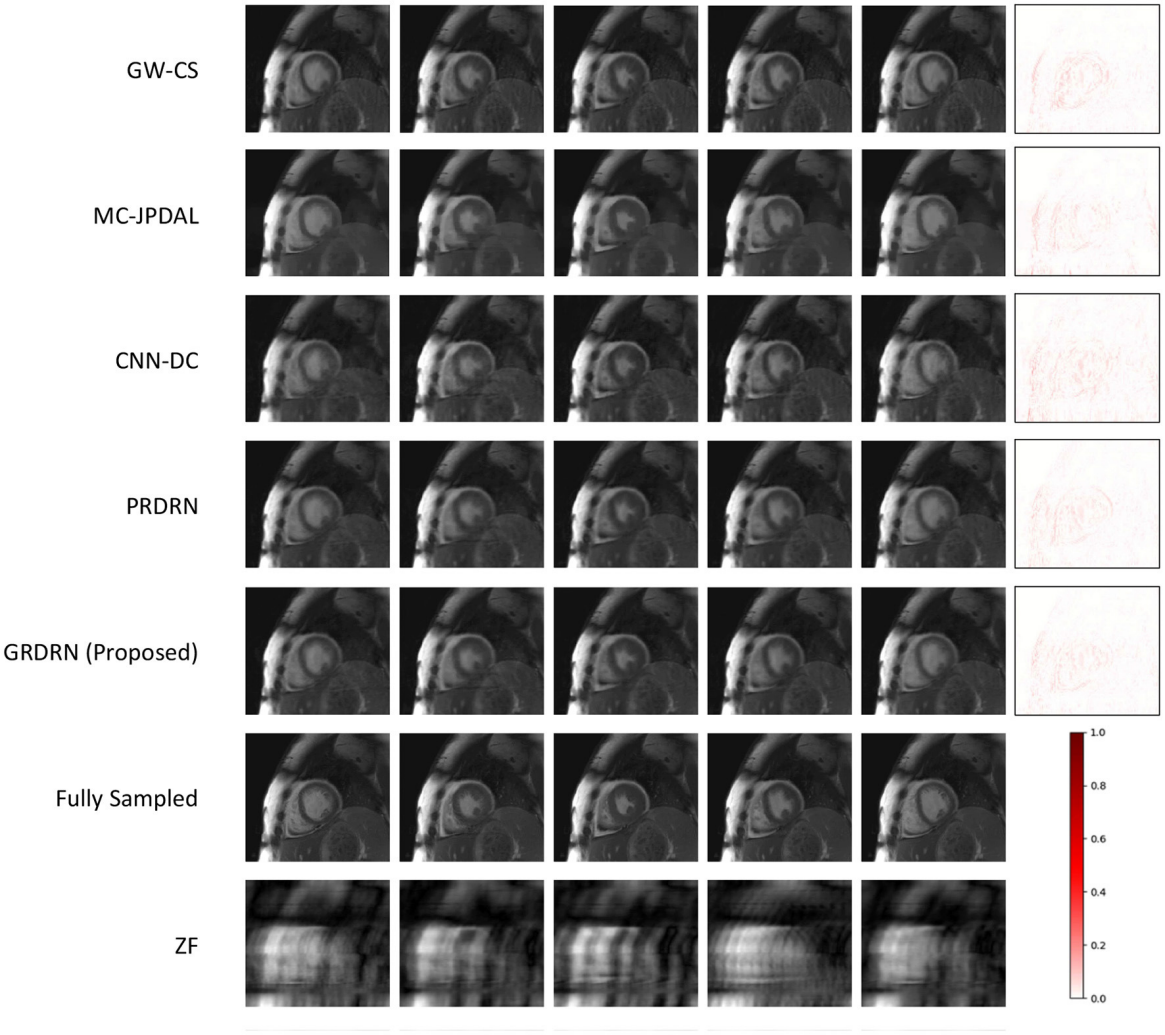
Visualization of reconstructed images as well as the fully sampled and zero-filling (ZF) reconstructed images for five representative frames (systolic to end-diastolic) in the case of acceleration rate 12×. The last column shows the difference maps between the reconstructed and ground-truth images for the frame in the fifth column. The color bar represents the magnitude of the error in the difference maps.

We have a total of 128 slices from 18 testing subjects to evaluate the reconstruction and motion estimation performance. Figure 3 shows the example reconstructions of GW-CS, MC-JPDAL, CNN-DC, PRDRN and the proposed GRDRN as well as the fully sampled and zero-filled reconstructed images for 12× acceleration, where five representative frames (frame 1, 6, 11, 16, and 21 ranging from systole to end-diastole) are demonstrated. The error map of frame 21 for each reconstruction method is shown in the last column to better visualize the reconstruction difference. Comparing between the reconstruction methods with ME/MC, over-smoothness and/or residual undersampling artifacts can be observed in MC-JPDAL and GW-CS reconstructions for this high acceleration factor, while the learning-based PRDRN and GRDRN performs better in removing artifacts and preserving image details. Quantitatively, the groupwise registration-based reconstruction approach GW-CS and GRDRN results in higher PSNR than the pairwise registration-based reconstruction method MC-JPDAL and PRDRN, respectively (GW-CS vs. MC-JPDAL: 36.89 vs. 31.46; GRDRN vs. PRDRN: 37.10 vs. 36.86), suggesting that groupwise registration works better than pairwise registration in improving the reconstruction quality. Comparing between the learning-based methods, more obvious residual undersampling artifacts can be observed in the CNN-DC reconstruction than PRDRN and GRDRN, indicating that incorporating motion estimation benefits reconstruction.

**Figure 4:**
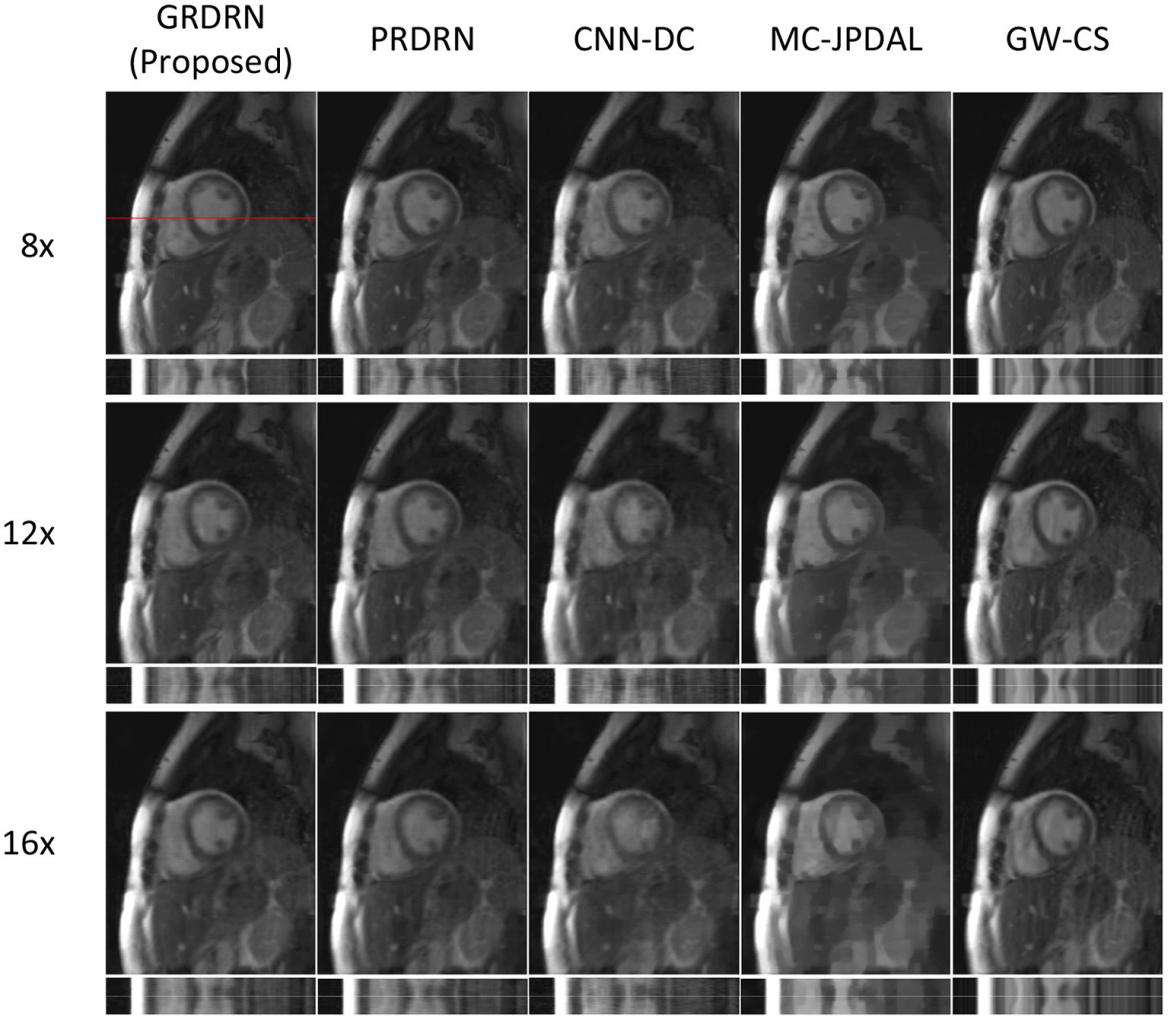
Visual comparison of reconstructions with different reconstruction approaches for 8×, 12×, and 16× acceleration rates. The temporal profile at the location indicated by the red line is shown for each reconstruction.

Figure 4 shows the representative cardiac cine images and temporal profiles reconstructed with the five reconstruction approaches for 8×, 12×, and 16× acceleration factors. The image quality of PRDRN and GRDRN degrades less than other reconstruction methods with the increasing of acceleration factors. The proposed GRDRN results in the best visual image quality for all acceleration factors among all the compared methods.

**Figure 5:**
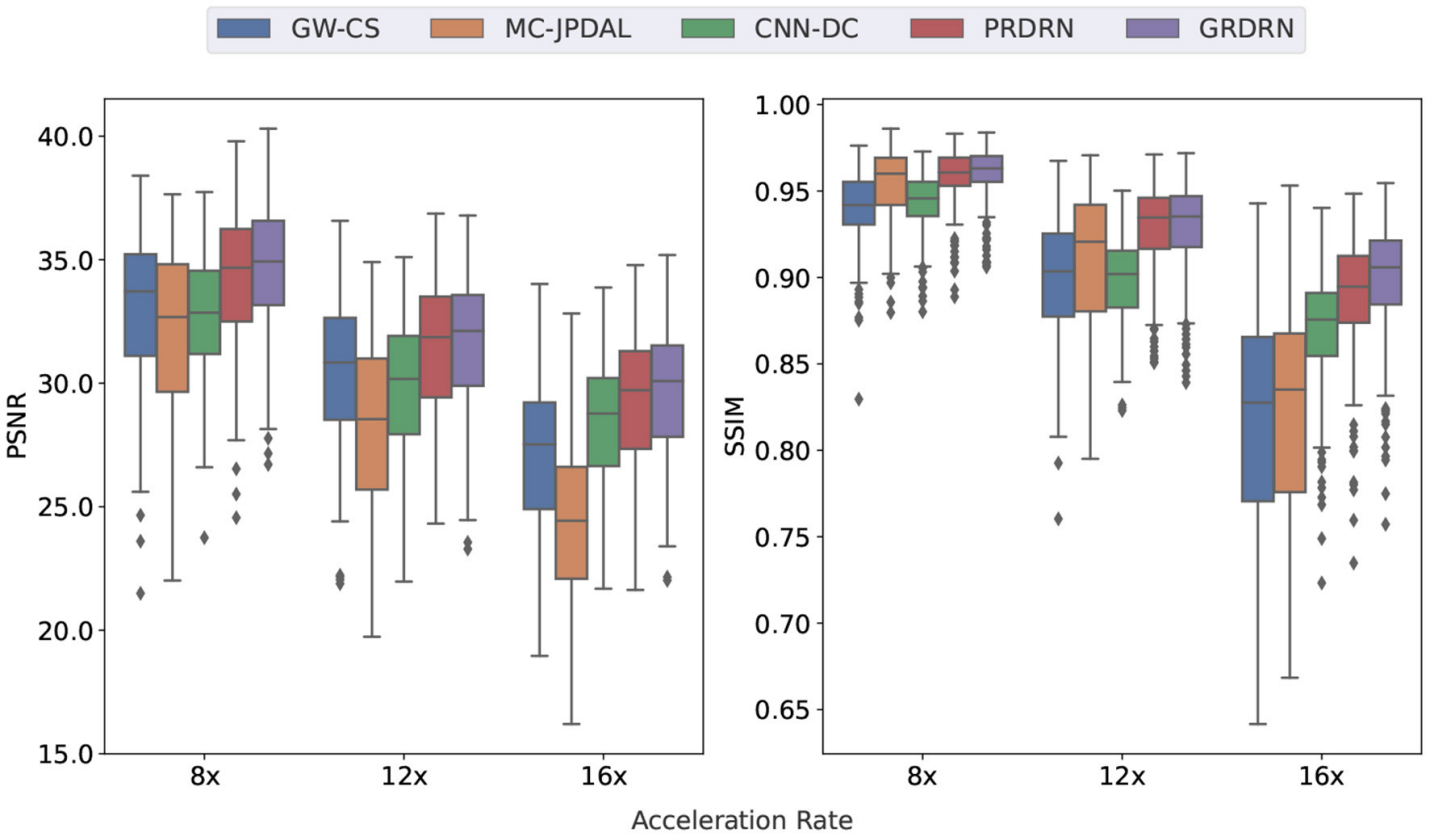
Box plots of PSNR and SSIM values of the reconstructed dynamic images with different reconstruction methods for 8×, 12×, and 16× acceleration rates. Boxes depict the 25 and 75% percentile, horizontal line shows the median, whiskers show the standard deviation and dots represent the outliers.

The PSNR and SSIM of all the five reconstruction methods for 8×, 12× and 16× accelerated cardiac cine MRI are shown in the box plots in Figure 5. The quantitative metrics agree with the visual assessment that the proposed GRDRN consistently performed the best among the testing methods. Notably, the PSNR and SSIM of GRDRN and PRDRN at 16× are similar to those of other reconstruction methods at 12×. We then emphasize the strength of the deep learning-based motion-estimating dynamic reconstruction approaches of producing good reconstruction quality even with high acceleration factors. The average computation times per slice of different reconstruction methods are: GRDRN 2.44s; PRDRN 2.38s; CNN-DC 0.29s; GW-CS 506.11s; and MC-JPDAL 298.59s. It can be seen that deep learning-based methods operate much faster than the traditional methods. Among the deep learning-based methods, CNN-DC is the fastest as it does not have the ME component, while the reconstruction times of GRDRN and PRDRN are similar.

### 3.2. Motion Estimation

**Figure 6:**
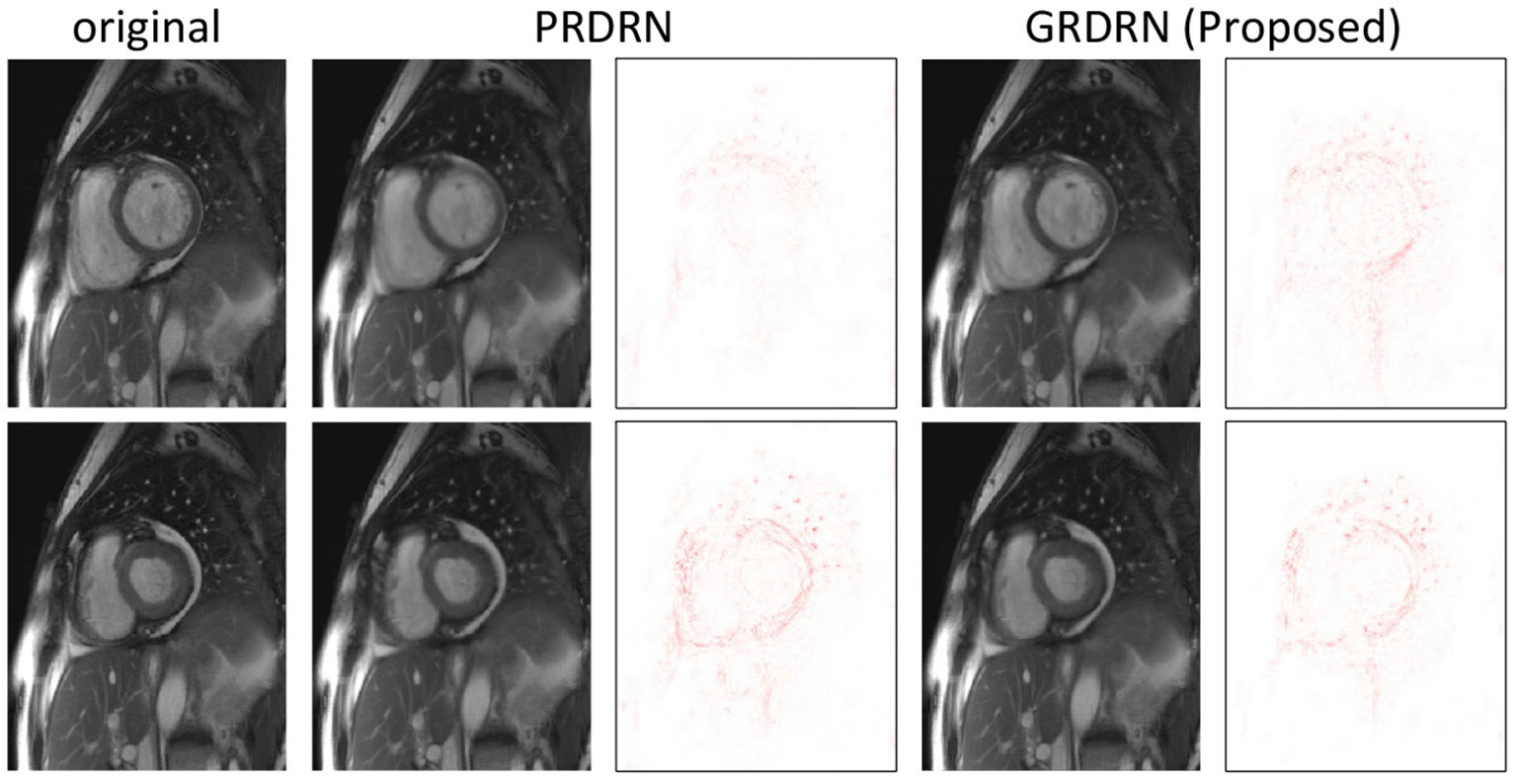
Visualization of generated dynamic images with motion fields estimated with PRDRN (pairwise registration) and proposed GRDRN (groupwise registration) models trained in 12× accelerated data. Two representative frames at diastole and systole are shown with the corresponding error maps.

**Figure 7:**
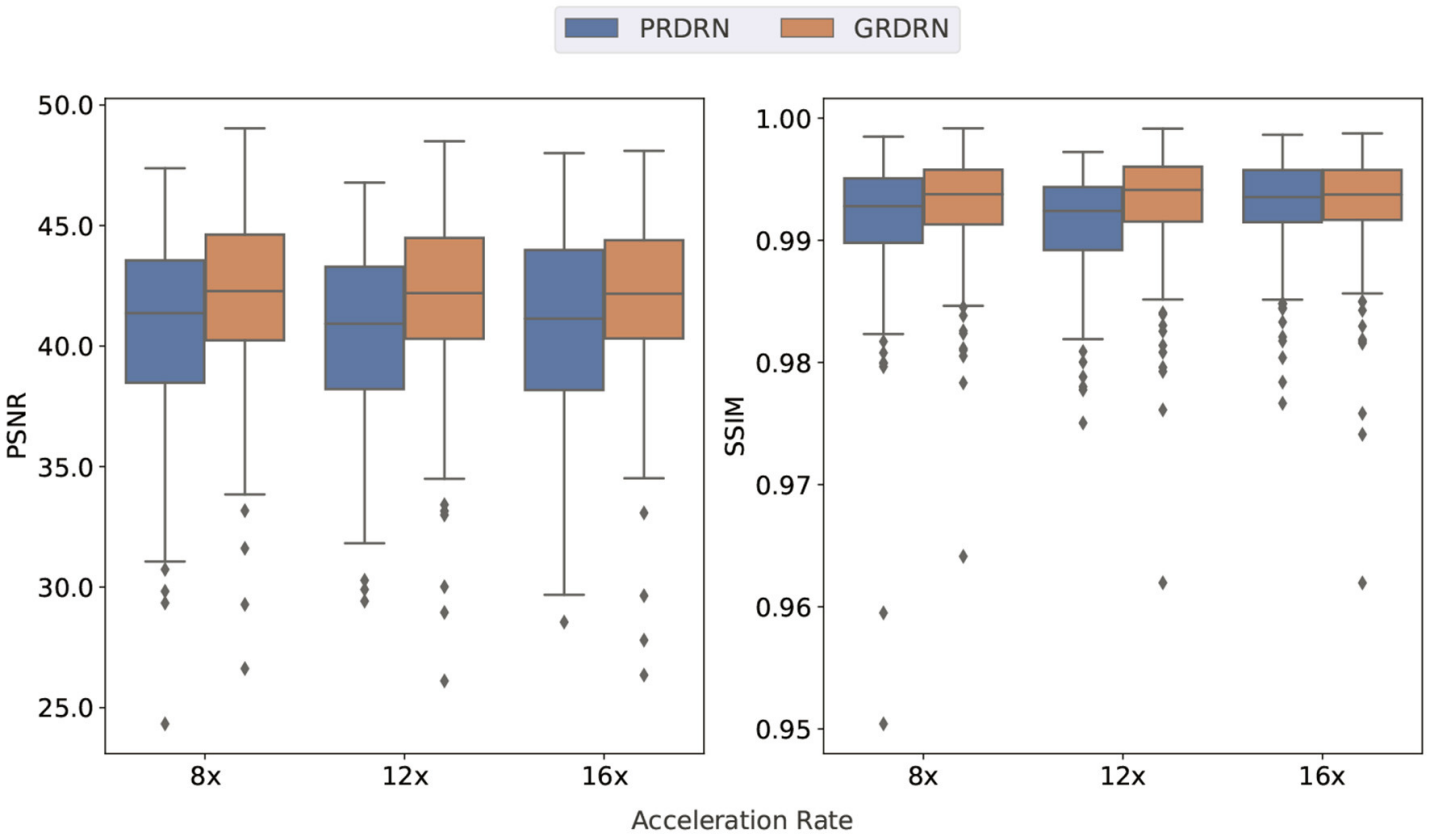
Box plots of PSNR and SSIM for the registration task with pairwise (PRDRN) and groupwise (GRDRN) registrations, where the metrics are reported between generated dynamic image sequences and the original ones.

Following the scheme described in the section of Evaluations, we evaluate the registration performance by assessing the dynamic images generated using the invertible motion fields. Animated images showing motion fields for a whole dynamic sequence with 8×, 12× and 16× accelerations are provided in Supplementary Figure S1. It is noted that the learned motion is smooth and reasonable, and is mostly in the cardiac region. Figure 6 illustrates two frames (one diastolic and one systolic frame) of the generated cardiac cine images with motion fields estimated with PRDRN and GRDRN models trained in 12× accelerated cardiac cine MRI. By visualizing the error maps, GRDRN achieves similar registration accuracy for both diastolic and systolic frames, while the error level of the systolic frame is obviously higher than the diastolic frame for PRDRN which uses the end-diastolic frame as the registration reference. Overall, the groupwise registration-based GRDRN has better registration than the pairwise registration-based PRDRN regarding PSNR (41.46 vs. 37.90) and SSIM (0.985 vs. 0.972) in this subject. Figure 7 provides the PSNR and SSIM of the generated cine images for 8×, 12×, and 16× acceleration factors. The higher PSNR of SSIM of GRDRN than PRDRN indicates the groupwise registration gives better motion estimation than pairwise registration, leading to better reconstruction of GRDRN as demonstrated in the previous section.

## 4. Discussion

We propose an end-to-end trainable joint learning approach which performs groupwise registration-based motion estimation and dynamic reconstruction, denoted as GRDRN. We construct an unrolled network architecture where the registration and reconstruction are optimized alternatively, and the two tasks are beneficial to each other as accurate motion estimation contributes to improving the reconstructed image quality and good image quality in turn helps to improve the motion estimation accuracy. We evaluate GRDRN in breath-hold cardiac cine MRI for aggressive undersampling rates of 8×, 12× and 16×, aiming to reduce the number of breath-holds substantially and ultimately to achieve whole-heart cine MRI in a single breath-hold. The proposed method consistently achieves improved reconstruction performance compared with deep learning dynamic reconstruction with pairwise registration PRDRN or without exploiting motion information CNN-DC, and the conventional state-of-the-art dynamic MRI reconstruction methods with ME/MC, confirming the superiority of GRDRN.

A common strategy for registration is to register a moving image to a reference image. When there are multiple images to be registered, another strategy that can be beneficial is to register multiple images to a common space instead of in pairs, the process of which is termed as groupwise registration. There are in general three different types of groupwise registration: sum-of-pairs approach that attempts to reduce the registration loss among all image pairs; reference-based approach that requires the designation of one image as reference; implicit template approach that implicitly determines the template image during registration, and can avoid the bias caused by selecting one particular image as reference while being more computationally efficient than the sum-of-pairs method (41). Deep learning groupwise registration has been adopted in several recent studies (36, 37, 42) and has demonstrated superior performance over pairwise registration. For example, Zhang et al. propose an one-shot learning groupwise registration network to register respiratory motion-resolved 3D CT images (36). Martín-González et al. (42) develop a deep learning framework to achieve groupwise registration of 2D dynamic sequence, in which the implicit template deep learning groupwise registration approach is adopted to estimate the nonrigid motion across the dynamic sequence.

The motion estimation performance is evaluated by employing the estimated invertible motion fields to generate a new sequence of cine images to be compared with the original dynamic images. The joint motion estimation and reconstruction model achieves similarly good quantitative metrics for a range of acceleration factors, indicating that the motion estimation in GRDRN is robust to undersampling artifacts. Moreover, the results indicate that groupwise registration performs better than pairwise registration in registering a set of dynamic images by finding a template image that lies in the geometric center of the group. Besides aiding in improving the reconstruction performance, cardiac motion estimation is an important step in myocardial strain analysis. The applicability of the estimated motion with the proposed joint learning approach to myocardial strain analysis will be investigated in the future work.

In GRDRN, motion-augmented dynamic images are generated based on both the intermediately reconstructed and the zero-filling reconstructed images as additional inputs to the reconstruction network. In our initial experiments, we have tried to use motion-augmented dynamic images generated from the intermediate reconstruction only, which leads to a decrease of the reconstruction PSNR of 1-2dB compared to reconstruction with both sets of motion-augments images. The possible reason could be that the undersampling artifacts are gradually removed in the unrolled iterations. However, the images of intermediate reconstructions tend to get smooth and may lose some fine details. On the other hand, the zero-filling motion-augmented images though being more undersampled, may contain more image details than the motion-augmented images generated from the intermediate reconstructions. Consequently, adding the zero-filling motion-augmented images will allow the reconstruction network to exploit such details at all stages of the cascade, and can ultimately improve the reconstruction performance. It is noted that similar strategy has been employed in a previous study (31) that adopts pairwise registration to augment the reconstruction.

The appearance of the heart and the motion pattern may be heterogeneous in the short-axis cardiac cine MR images from the base to the apex of the heart, which may lead to heterogeneous reconstruction performance for the basal, middle and apical slices. We then analyze the 128 testing slices thoroughly and find that reconstruction and motion estimation metrics of apical slices are similar to those of middle slices. However, for some basal slices where the myocardium is not intact we do observe the performance drop for all the reconstruction methods, indicating the basal slices are more challenging to be reconstructed. Specifically, there is a total of 17 basal slices, and the reconstruction PSNR of 8× accelerated cine MRI of the basal slices is 32.46 ± 3.46, 31.84 ± 3.24, 30.89 ± 2.94, 30.81 ± 3.34, and 30.06 ± 4.08 for the methods of GRDRN, PRDRN, CNN-DC, GW-CS and MC-JPDAL respectively, compared with PSNR of 34.82 ± 2.73, 34.19 ± 2.94, 32.90 ± 2.44, 33.30 ± 3.00, and 32.34 ± 3.29 of non-basal slices. We can see that the proposed GRDRN still outperforms other testing methods in the challenging basal slices.

The single-coil acquisition scenario is simulated in this work to reduce computation complexity and memory consumption, while the proposed GRDRN can be extended to multi-coil reconstruction by adapting the data consistency layer. It is noted that the applicability of the proposed method needs to be further tested in prospectively undersampled data.

In conclusion, we propose an end-to-end trainable joint learning approach which performs groupwise registration-based motion estimation and dynamic reconstruction. The groupwise registration network GRN, predicts invertible motion fields between all dynamics and an implicit template. Taking advantage of the estimated motion, all measurements along the temporal dimension are fused to the implicit template, from which a new sequence of dynamic images with lower undersampling can be generated to assist in the reconstruction. We evaluate the proposed approach on cardiac cine MRI datasets for aggressive acceleration factors and demonstrate that the proposed GRDRN can achieve state-of-the-art reconstruction performance benefiting from the motion information from the groupwise registration.

## Data Availability Statement

The original contributions presented in the study are included in the article/Supplementary Materials, further inquiries can be directed to the corresponding author/s.

## Author Contributions

The manuscript was written by HQ and revised by JY, TK, PH, and PL. The study was designed by JY and HQ. The experiments were performed by JY. Research funds were offered by HQ. All authors contributed to the article and approved the submitted version.

## Funding

This work was supported in part by the National Natural Science Foundation of China under Grant No. 82102027.

## Conflict of Interest

The authors declare that the research was conducted in the absence of any commercial or financial relationships that could be construed as a potential conflict of interest.

## Publisher's Note

All claims expressed in this article are solely those of the authors and do not necessarily represent those of their affiliated organizations, or those of the publisher, the editors and the reviewers. Any product that may be evaluated in this article, or claim that may be made by its manufacturer, is not guaranteed or endorsed by the publisher.

## Supplementary Material

Supplementary Figure S1 Animated visualization of generated motion fields estimated by PRDRN and GRDRN for a whole dynamic sequence with 8x, 12x and 16x accelerations.
